# Amoebic Hepato-Pericardial Fistula Complicating Amoebic Liver Abscess Treated With Pericardiotomy: A Case Report

**DOI:** 10.7759/cureus.28262

**Published:** 2022-08-22

**Authors:** Hamada Alsheikh, Nour Shaheen, Ahmed Shaheen, Saleh Raslan, Mostafa Meshref, Yara Amro, Sarya Swed, Abdulqadir J Nashwan

**Affiliations:** 1 Cardiology, Al-Azhar University, Cairo, EGY; 2 Internal Medicine, Alexandria University, Alexandria, EGY; 3 Medicine, Faculty of Medicine, Alexandria University, Alexandria, EGY; 4 Cardiology, Hail Cardiac Center, Hail, SAU; 5 Neurology, Al-Azhar University, Cairo, EGY; 6 Pharmacy, Ministry of Health, Cairo, Cairo, EGY; 7 Medicine, Aleppo University, Aleppo, SYR; 8 Qatar University (QU) Health, Qatar University, Doha, QAT; 9 Nursing, University of Calgary in Qatar, Doha, QAT; 10 Nursing, Hamad Medical Corporation, Doha, QAT

**Keywords:** constrictive pericarditis, cardiac tamponade, pericardial effusion, liver abscess, amoeba

## Abstract

Parasitic infections like amoebiasis are often asymptomatic in the tropics, but the invasive disease can cause an amoebic liver abscess. During pericardiocentesis, amoebiasis is more noticeable in left lobe abscesses with chocolate-like pus drainage. Here, we present an unusual amoebic liver abscess that erupted into the pericardial cavity via a diaphragmatic fistula. An emergency pericardiotomy was performed to relieve cardiac tamponade, and the liver abscess was evacuated through a diaphragmatic rent identified during the surgery. This illustrates the catastrophic complications of an amoebic liver abscess.

## Introduction

Amoebiasis is a common disease worldwide that results from *Entamoeba histolytica*. It is usually asymptomatic and colonizes the gastrointestinal tract. Amoebiasis is asymptomatic in 90% of patients, although it can present outside the gastrointestinal tract (GIT) in rare cases as frank dysentery to abscesses of the liver. An amoebic liver abscess (ALA) is considered the most common extraintestinal manifestation of an *E. histolytica* infection [1]. If not recognised and treated early, ALA can burst and damage the nearby organs. Depending on the rupture location, the symptoms might range from moderate sickness to catastrophic organ failure. Even though it is rare, amoebic pericarditis is the most dangerous complication of amoebic liver abscess. This complication occurs more frequently in patients with an amoebic liver abscess in the left lobe superior surface [2]. We present a case of an amoebic hepato-pericardial fistula aggravating an amoebic liver abscess of the left lobe, which resulted in the pericardial effusion and cardiac tamponade.

## Case presentation

**Figure 1:**
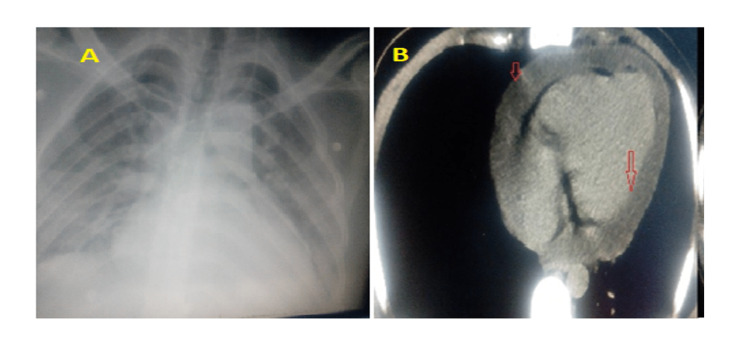
(A) CXR shows cardiomegaly and clear lungs. (B) CT chest showing massive circumferential pericardial effusion (red arrows).

A 28-year-old male was healthy before attending the emergency department. He had a history of laparoscopic cholecystectomy three months ago. He presented with pleuritic chest pain, dyspnoea, and orthopnoea. ECG showed signs of pericarditis with knuckle sign, complete routine labs, CXR (Figure 1A), and echocardiography study showed moderate pericardial effusion. The patient was admitted to the hospital to screen for the underlying cause. Echocardiography revealed massive pericardial effusion with no signs of tamponade. However, within a few hours, his situation rapidly deteriorated, he collapsed, and clinical signs of tamponade were evident. ECG, CXR, and echo-Doppler studies were repeated in the cardiac care unit (CCU). At this time, the patient was diagnosed with cardiac tamponade. A fluid collection was also reported below the diaphragm; therefore, CT chest (Figure 1B), pelvic, and abdominal ultrasound were done. The CT showed a massive pericardial effusion and an abscess-like cavity in the left lobe of the liver.

**Figure 2:**
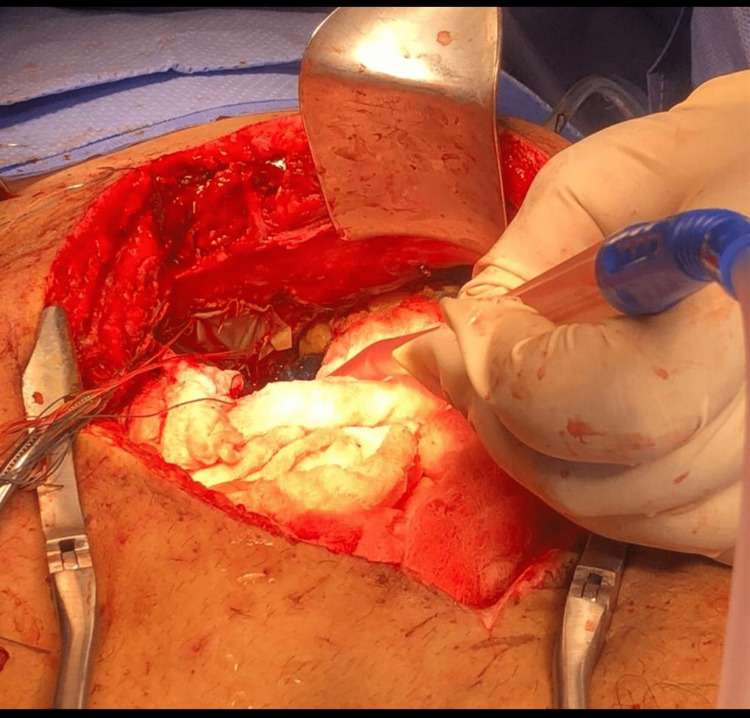
Surgical drainage through the diaphragm.

The patient was taken to the catheterisation laboratory, where pericardiocentesis was performed. The attending physician noted a chocolate-coloured "anchovy paste" consistency pus was drained during pericardiocentesis. The surgeons, cardiac and general surgeons, were immediately consulted, and the patient was transferred to the operating room (OR). The patient was diagnosed with a hepatic abscess in the left lobe, complicated by a fistula that opened into the pericardium in the OR. This was deemed the cause of the patient's cardiac tamponade; drainage and lavage were completed (Figure 2). A few days later, the fistula was closed. Follow-up echocardiography was performed daily and weekly with CT chest scans for one month. The patient was discharged with regular follow-up appointments scheduled while assessing if the patient should undergo a pericardiotomy. Constrictive pericarditis was suspected due to thickening in the pericardium seen on echocardiography and a CT chest scan a month post-surgery. The analysis of the drained material matched the clinical scenario, and the suspicion of the amoebic liver abscess was confirmed. The final diagnosis was an amoebic liver abscess complicated by a hepato-pericardial fistula causing cardiac tamponade. The gastroenterologist recommended commencing IV metronidazole 400 mg three times a day (TID) for one week, followed by oral metronidazole for several weeks and other supportive medications.

## Discussion

Amebiasis is an uncommon cause of pericarditis that often manifests as a liver abscess. It occurs more frequently in tropical regions and is the leading cause of parasite death globally. Even while an invasive illness is usually asymptomatic, if it goes untreated, it may develop and cause several complications. A rupture into the thoracic (pleural or pericardial cavities) or abdominal (peritoneal cavities) areas is an uncommon but harmful consequence [3]. A 16-month-old infant with amoebic liver perforation in the pleural, pericardial, peritoneal, and gastric regions has been documented [4]. Various case series feature a substantial number of hepatic amoebic abscess patients. One study, conducted over the course of more than 18 years, looked at 501 cases of amoebic liver abscess [2]. Five hundred three amoebic liver abscess cases were documented over 21 years in another large case series from China. This series saw a perforation-related complication in 22% of patients [5]. The pericardial cavity perforation complication is still very uncommon.

Similar cases of reported pericardial rupture of ALA developing cardiac tamponade were often treated with pericardiotomy. Percutaneous drainage alone is also used to treat intra-pericardial rupture when the development of cardiac tamponade does not hinder emergency intervention. There are four different varieties of amoebic pericarditis: suppurative (purulent), non-suppurative (non-purulent), constrictive, and hydropneumopericardium [6]. Some patients continue to pass away of unknown causes even after receiving a quick diagnosis and treatment with pericardial aspiration or pericardiotomy. Theoretically, cardiac arrhythmia-related sudden death is caused by ultra-microscopic alterations in the myocardium [7]. These changes include cellular and mitochondrial transient abnormalities such as mitochondrial swelling, glycogen loss, and cytosol swelling, which are related to arrhythmia-induced myocardial hypoxemia that is triggered by the parasite’s toxins. Before the patients' death from ventricular fibrillation, a variety of tachyarrhythmias and bradyarrhythmias were documented in the critical care unit during a period when they seemed to be developing well. Our patient was thought to have type 2 pericarditis with a chance of developing type 3. Patients who survive the second stage may develop constrictive pericarditis, though it is rarely described in the literature. Since 1790, when Equiay Moro reported the first case of amoebic pericarditis, a small number of constrictions have been reported. Lamont and Pooler reported that the frequency of this complication was about 2.8% over 50 years ago [8]. However, such cases have been reported less often in the last decade due to early diagnosis and the timely institution of effective amoebicidal drugs.

Macleod reported seven cases of constrictive pericarditis in his series of 25 cases of amoebic pericarditis [9]. In 250 cases of reported hepatic amoebiasis, Lamont identified seven cases of pericardial involvement. Three of them developed constrictive pericarditis, and all died despite the treatment given [8]. This stage is likely to be confused with tubercular pericarditis (TB), the most common cause of constrictive pericarditis in the developing world. However, as opposed to months as in TB, the development of constriction in amoebiasis occurs more quickly over the course of weeks. The stage of effusion in amoebic pericarditis also blends immediately into the constriction phase, in contrast to tubercular pericarditis, where it is followed by a quiet period before the commencement of constriction. Recently, there have been fewer amoebic pericarditis occurrences, probably because the amoebic liver abscess is now being treated early and less often, reducing the risk of developing this unusual complication. There is evidence in the literature that this stage of early constriction, as shown here in our case, may resolve with conservative management. Though others argue that once this complication occurs, pericardiectomy is the treatment of choice.

## Conclusions

This case report describes a rare and difficult-to-diagnose cause of cardiac tamponade. Our patient was asymptomatic, with the presentation solely occurring due to the pericardial effusion. Physicians must be cautious regarding pericardial complications of a large amoebic left lobe liver abscess. A liver abscess determines amoebic pericarditis, the high serum level of amoebic antibodies, and the presence of *E. histolytica* parasites in pericardial aspirate and the drained liver abscess. These are strong determinants of impending constrictive amoebic pericarditis even in the absence of suppuration pericardial effusion in the presence of ALA and the high serum of antibodies. That urges the commencement of prolonged and extensive amoebicidal drugs in addition to corticosteroids, as well as close follow-up for early detection of cardiac constriction.
